# Theoretical and Experimental Considerations for a Rapid and High Throughput Measurement of Catalase In Vitro

**DOI:** 10.3390/antiox11010021

**Published:** 2021-12-22

**Authors:** Ouardia Bendou, Ismael Gutiérrez-Fernández, Emilio L. Marcos-Barbero, Nara Bueno-Ramos, Ana I. González-Hernández, Rosa Morcuende, Juan B. Arellano

**Affiliations:** Department of Abiotic Stress, Institute of Natural Resources and Agrobiology of Salamanca (IRNASA-CSIC), Cordel de Merinas, 40–52, 37008 Salamanca, Spain; ouardia.bendou@irnasa.csic.es (O.B.); ismael.gutierrez@irnasa.csic.es (I.G.-F.); emiliol.marcos@irnasa.csic.es (E.L.M.-B.); nara.bueno@irnasa.csic.es (N.B.-R.); ana.gonzalez@irnasa.csic.es (A.I.G.-H.); rosa.morcuende@irnasa.csic.es (R.M.)

**Keywords:** catalase, hydrogen peroxide, microplate reader, polynomial fitting, suicide substrate

## Abstract

A rapid and high throughput protocol to measure the catalase activity in vitro has been designed. Catalase is an enzyme with unusual kinetic properties because it does not follow the standard Michaelis–Menten model and is inactivated by H_2_O_2_. This makes the analysis of the two rate equations of the second-ordered reactions of the kinetic model rather complex. A two-degree polynomial fitting of the experimental data is proposed after transforming the exponential form of the integrated rate equation of the [H_2_O_2_] into a polynomial using the Taylor series. The fitting is validated by establishing an experimental linear relationship between the initial rate of the H_2_O_2_ decomposition and the protein concentration, regardless of the suicide inactivation that catalase might undergo beyond *t* > 0. In addition, experimental considerations are taken into account to avoid statistical bias in the analysis of the catalase activity. ANOVA analyses show that the proposed protocol can be utilized to measure the initial rate of the H_2_O_2_ decomposition by catalase in 32 samples in triplicates if kept below 8 mM min^−1^ in the microplate wells. These kinetic and statistical analyses can pave the way for other antioxidant enzyme activity assays in microplate readers at small scale and low cost.

## 1. Introduction

Catalase is an enzyme that has amazed life science and biomedical researchers since 1900, when Loew [1] coined the name catalase for the enzyme capable of catalyzing H_2_O_2_ decomposition (here termed catalytic activity). This reactive oxygen species (ROS) diffuses almost freely through membranes and puts living organisms in jeopardy when it forms the strongly noxious HO^●^ radical in the Fenton reaction. Remarkably, H_2_O_2_ is also a redox signaling molecule [2]. Because of this H_2_O_2_ duality, catalase is essential to ensuring redox balance in aerobic and facultative (micro)organisms [3], where H_2_O_2_ is produced as a by-product of aerobic respiration. Evidence for the presence of catalase in some strictly anaerobic microorganisms has also been put forward [4]. Together with its catalytic activity, another activity (termed peroxidatic activity) occurs when H_2_O_2_ concentration is at low level and other hydrogen donors replace H_2_O_2_ in the second stage (see below) of the enzyme reaction [5]. Interestingly, both catalase activities have found notable applications in industrial biotechnology and remediation of environmental pollutants [6]. Nowadays, the enzyme is still said to be veiled in mystery because catalase can also (*i*) act as an oxidase, (*ii*) produce ROS under UV radiation, (*iii*) reduce free NADPH to prevent inactivation and (*iv*) be regulated by post-translational modification by ROS, reactive nitrogen species and even H_2_S [7,8]. Moreover, catalase also shows high thermostability [9].

The most common method for measuring the catalase activity in vitro is spectrophotometric, where the H_2_O_2_ decomposition is followed in the UV region [5,10]. Specifically, catalase performs the dismutation reaction of H_2_O_2_ in two stages following a ping-pong mechanism [11,12]. In the first stage (Reaction 1), a molecule of H_2_O_2_ oxidizes the heme iron group of the enzyme to yield an oxyferryl group and a cationic radical of porphyrin $\left[{\mathrm{Por}}^{+●}-{\mathrm{Fe}}^{\mathrm{IV}}=O\right]$, while a molecule of H_2_O is released into the medium. This intermediate state of catalase is denoted Compound I. In the second stage (Reaction 2), a second molecule of H_2_O_2_ reduces Compound I, causing the enzyme to return to its initial state, while simultaneously a second molecule of H_2_O and a molecule of O_2_ are produced.


(R.1) $$\mathrm{Catalase}+H_{2}O_{2}\overset{k_{I}}{\to }\mathrm{Compound}\;I+H_{2}O\;$$

(R.2) $$\mathrm{Compound}\;I+H_{2}O_{2}\overset{k_{\mathrm{II}}}{\to }\mathrm{Catalase}+H_{2}O+O_{2}.$$

With particular regard to its kinetics, catalase is a unique enzyme because it does not follow the standard scheme of the Michaelis–Menten model [5,11]. Firstly, it is not possible to saturate the enzyme by substrate concentration within experimental conditions. Secondly, catalase can be inactivated by its own substrate H_2_O_2_ (i.e., suicide substrate inactivation). Particularly, compound I can be converted into Compound II $\left[\mathrm{Por}-{\mathrm{Fe}}^{\mathrm{IV}}=O\right]$ in a one-electron reaction, which can then be converted into Compound III after a two-electron reaction with H_2_O_2_ [13]. These one- and two-electron reactions can reversibly or irreversibly inactivate catalase (Reactions 3–5). The suicide substrate inactivation of catalase and other enzymes with catalase-like activity is still a matter of great interest to industrial biotechnology [9,14].

(R.3) $$\mathrm{Compound}\;I+e^{-}\to \mathrm{Compound}\;\mathrm{II}\;(inactive)\;$$

(R.4) $$\mathrm{Compound}\;\mathrm{II}+e^{-}\to \mathrm{Catalase}\;(active)\;$$

(R.5) $$\mathrm{Compound}\;\mathrm{II}+H_{2}O_{2}\to \mathrm{Compound}\;\mathrm{III}\;(inactive)\;.$$

Most of the kinetic models used to follow the catalase activity are based on the measure of the overall rate of H_2_O_2_ decomposition imposed by the limiting step of the reaction between Compound I and H_2_O_2_ [11]. If, for instance, H_2_O_2_ is present at concentrations low enough to disregard enzyme inactivation and also the measure of the initial rate of the H_2_O_2_ decomposition is determined in the first tens of seconds, then the process can be described as a first-order reaction. In this first kinetic model, only the forward rate constants (*k*_I_ and *k*_II_) of the two stages are considered. Other kinetic models take into account the enzyme inactivation; therefore, the kinetic system is composed of two-second order reactions. Here, the experimental traces of the H_2_O_2_ decomposition are recorded for several (tens of) minutes. To solve the system of the differential equations of this second kinetic model (see the section of Results and Discussion), the kinetic system only considers the forward rate constants of the concurrent H_2_O_2_ decomposition and enzyme inactivation reactions under non-steady state conditions [15]. The integrated rates of the differential equations are rather complex and the non-linear fitting of the experimental traces requires significant time-consuming computer analysis.

We introduce a practical, rapid, and high-throughput spectrophotometric protocol to measure the catalase activity in vitro in microplate readers, when the use of a liquid handling robot is not affordable [16]. The researchers can deal simultaneously with multiple technical and biological replicates of samples holding a wide range of initial rates for the catalase activity. The protocol also makes use of an easy polynomial fitting procedure to determine the initial rates under experimental conditions regardless of the suicide substrate inactivation that catalase might undergo during the kinetic reading.

## 2. Materials and Methods

### 2.1. Computational Modeling

The computer algebra system Wolfram Mathematica v. 12.2 (Champaign, IL, USA) [17] was used to program scripts to solve the system of the first-order ordinary differential equations (ODEs) of the kinetic model of catalase, where it undergoes suicide substrate inactivation. The integrated analytical solutions for the rates of the H_2_O_2_ decomposition and the enzyme inactivation were obtained after considering the reverse reactions to be negligible. The values for the kinetic rate constants and the concentrations of the reactants were chosen to illustrate the (mis)match between the exponential time-dependence function of the reactant concentration (i.e., [H_2_O_2_]) and the one-degree and two-degree polynomial functions obtained using the Taylor series as time approaches zero. More details are included in the Results and Discussion section.

### 2.2. Plant Material

Flag leaves of the main stem of spring wheat (*Triticum aestivum* L. cv. Gazul; GnplS accession number 20351, https://doi.org/10.15454/DO8TC0, 11 November 2021) were used in the present study. At ear emergence, the leaves were harvested under the same growth conditions described in the study by Marcos-Barbero et al. [18]. They were detached by cutting through the base of the petiole and immediately submerged in liquid N_2_, and preserved at −80 °C until analysis [19]. An amount of approximately 1 g of frozen leaf tissue was ground using a pre-cooled mortar and pestle with liquid N_2_ and the fine powder transferred into microfuge tubes and kept at −80 °C until the catalase assay was carried out. Several batches of flag leaves were used and the grinding procedure was repeated as many times as necessary.

### 2.3. Enzyme Activity Assay

The catalase activity was measured following the spectrophotometric method described by Aebi [5] with the modifications introduced by Pérez-López et al. [20]. Other modifications are described below. In the last step of the extraction procedure, the outlet volumes from the Sephadex^TM^ G-25 superfine columns (Merck, Kenilworth, NJ, USA), i.e., 180 µL per column, of 24 leaf extractions were collected, grouped and, finally, split into seven sample fractions. The sample fractions formed a linear gradient from low to high total protein concentration after the dilution with the appropriate volume of the extraction buffer containing 0.1 mM EDTA, 0.1 mM phenylmethanesulfonyl fluoride, 2 mM dithiothreitol, 0.2% (*v*/*v*) Triton X-100, 50 mM Tris-HCl pH 7.8. An eighth fraction was added as a blank. The enzyme assay was carried out in 50 mM KH_2_PO_4_/K_2_HPO_4_ pH 7.0 at 25 °C (hereafter assay buffer). Two different protocols were followed. In the first (here termed standard protocol), there was only one assay buffer containing 25 mM H_2_O_2_. In the second (here termed proposed protocol), the assay buffer was split into two halves: one free of H_2_O_2_ and another containing the right concentration of H_2_O_2_ to reach a consistent final concentration of 25 mM H_2_O_2_ in the assay mixture at the beginning of the enzyme activity assay. The value of 43.6 M^−1^cm^−1^ for the extinction coefficient of H_2_O_2_ at 240 nm in water was used for calculations. The total protein concentration ranged between 0 and 100 μg mL^−1^ in the assay mixture. The protein concentration was measured using the Bradford protein assay. Linearization of the protein assay was achieved using the ratio of absorbance at 590 nm and 450 nm [21]. Bovine serum albumin was used as the standard protein. All the chemicals were purchased from Merck (Kenilworth, NJ, USA).

A multimode microplate reader FLUOstart^®^ Omega (BMG Labtech, Ortenberg, Germany) was used to monitor the H_2_O_2_ decomposition by catalase at 240 nm. The main technical features of the microplate reader (i.e., heating system, software, etc.) were described in the study by Mellado-Ortega et al. [22]. In the standard protocol, an aliquot of 5–10 μL from each of the sample fractions were deposited at the bottom of the wells. The selected volume depended on the batch of the flag leaf powder used. Then, a volume of 295–290 μL of the assay buffer containing 25 mM H_2_O_2_ was added to the wells. After this addition, the 96-well plate was briefly shaken for 5 s and then the kinetic reading started immediately. In the proposed protocol, an aliquot of 5–10 μL from each of the sample fractions and a volume of 195–190 μL of the H_2_O_2_-free assay buffer were added to the wells and the 96-well plate was pre-shaken for 30 s to ensure homogeneity. Then, 100 μL of the second half of the assay buffer containing H_2_O_2_ was added to the wells. After this last addition, the 96-well plate was also briefly shaken for 5 s and then the kinetic reading started immediately, as described for the standard protocol. The kinetic reading mode followed a vertical zig-zag pattern starting in plate position A1. Corning^®^ 96-well acrylic microplates with UV transparent flat bottom were used. The optical path was calculated to be 0.84 cm when using a final volume of 300 μL in the wells. Linear and two-degree polynomial fittings of the experimental data points were performed using the MARS data analysis software (version 3.02 R2) supplied by BMG Labtech (Ortenberg, Germany). The values for the r-squared were above 0.99 for the two-degree polynomial fitting of the experimental data showing curvilinear trend. No attempts beyond a two-degree polynomial fitting are included in the study. The reason for using a two-degree polynomial for the fitting is explained in the Results and Discussion section.

### 2.4. Statistical Analysis

In the present study, two independent experiments were carried out to establish whether the proposed protocol (to be implemented in microplate readers) represented a real advance in the analysis of the catalase activity in vitro. In the first statistical analysis, the reduction of the loading time of the assay buffer containing H_2_O_2_ was analyzed (Experiment I). In the second statistical analysis, the dependence of the measured values for the initial rate of the H_2_O_2_ decomposition by catalase on the well position was inspected (Experiment II).

A total of 480 samples (i.e., 2 × 30 × 8, protocols × replicates × lanes) were inspected to carry out the statistical analysis of Experiment I. The number of technical replicates for each of the eight sample fractions were 3, 6, 9 or 12 (i.e., 30 in total) for four different trials. For example, twelve replicates corresponded with a trial where all the 96 wells of the microplate were filled in (i.e., 8 × 12). The set-up for Experiment I is shown in the supplementary material (Figure S1) together with both the loading time for the assay buffer containing H_2_O_2_ and the reading time per cycle for the four different trials.

A total of 480 samples (i.e., 3 × 8 × 4 × 5, replicates × lanes × blocks × repetitions) were used in the statistical analysis of Experiment II. In this analysis, the proposed protocol was used in order to minimize the loading time of the assay buffer containing H_2_O_2_. The set-up for this second experiment is shown in Figure S2. The position of the blocks fixes the inherent kinetic reading time per cycle of the microplate reader.

The statistical analysis of the two independent experiments started evaluating the assumptions that the ANOVA analyses make about the data. The package stats from R were utilized: (*i*) to identify outliers, (*ii*) to check the normal distribution of levels within factors using the Shapiro–Wilk test, (*iii*) to assess the homogeneity of variance across levels using the Levene test, (*iv*) to explore interaction between factors using two-way ANOVA analyses, and (*v*) to perform pairwise comparisons between levels with the Bonferroni correction [23].

## 3. Results and Discussion

### 3.1. Theoretical Considerations for an Easy Fitting of the Experimental Data of the H_2_O_2_ Decomposition by Catalase with a Two-Degree Polynomial

The Michaelis–Menten equations for the initial rate of an enzyme-catalyzed reaction with one substrate or multiple substrates are elegantly derived from the ordinary differential equations (ODEs) describing their kinetic models after accepting several boundary conditions [24]. The steady state approximation for the concentration of the intermediates of the reaction is widely applied and its use implies that they will reach saturation rapidly, particularly when [*A*]*_o_* >> [*E*]*_o_* at the initial time (*t* = 0), and then they will (be accepted to) remain in steady state for a considerable time. [*A*] and [*E*] stand for the concentrations of the enzyme substrate(s) and the enzyme, here H_2_O_2_ and catalase, respectively. The steady state approximation is ruled out when dealing with the catalase because it undergoes suicide substrate inactivation and the intermediates do not reach saturation. In this regard, Feuers et al. [15] presented a kinetic model under non-steady state conditions, where the overall second-order reactions of both the H_2_O_2_ decomposition by catalase (Equation (1)) and the suicide (and irreversible) inactivation of catalase by H_2_O_2_ (Equation (2)) were put together in the system of ODEs (shown below), where $k_{1}$ and $k_{2}$ stand for the overall forward rate constants of the second-order reactions.

(1) $$-\frac{d[A]}{dt}=k_{1}[E][A]$$

(2) $$-\frac{d[E]}{dt}=k_{2}[E][A]\;.$$

In this kinetic model, the reverse rate constants and the concentration of the reaction intermediates were set equal to zero. The integrated rate equations obtained by Feuers et al. [15] can be written in different ways and among all of them we have chosen the following:

(3) $$[A]=\frac{{\left[A\right]}_{o}\left(k_{2}{\left[A\right]}_{o}-k_{1}{\left[E\right]}_{o}\right)}{k_{2}{\left[A\right]}_{o}-k_{1}{\left[E\right]}_{o}\times Exp\left[-\left(k_{2}{\left[A\right]}_{o}-k_{1}{\left[E\right]}_{o}\right)t\right]}$$

(4) $$[E]=\frac{{\left[E\right]}_{o}\left(k_{2}{\left[A\right]}_{o}-k_{1}{\left[E\right]}_{o}\right)}{k_{2}{\left[A\right]}_{o}\times Exp\left[\left(k_{2}{\left[A\right]}_{o}-k_{1}{\left[E\right]}_{o}\right)t\right]-k_{1}{\left[E\right]}_{o}}\;.$$

If it were assumed there was no suicide substrate inactivation by H_2_O_2_ (i.e., *k*_2_ = 0), the first integrated rate equation could be reduced to that of a first-order reaction and the second to [*E*] = [*E*]*_o_*. Equation (3) is used (after some transformations) to fit the experimental data and to obtain the initial rates for the H_2_O_2_ decomposition by catalase and the enzyme inactivation by H_2_O_2_ [15]. Alternatively, one can follow—as we describe here—a shortcut if the main goal of the study is just to know the initial rate for the H_2_O_2_ decomposition. Equations (3) and (4) are infinitely differentiable, therefore they can be transformed into a sum of integer powers of polynomials using the Taylor series to calculate approximately [*A*] (and also [*E*]) around a defined value for *t*. On doing this, the approximate value for [*A*] increasingly approaches its correct value as the degree of the polynomial augments. Here, if our attention is only focused on [*A*] and its function is only expanded into a polynomial of degree two (for the sake of clarity) around *t* = 0, the Taylor series expansion yields a polynomial whose coefficients are as follows:

(5) $$[A]\approx {\left[A\right]}_{o}-k_{1}{\left[E\right]}_{o}{\left[A\right]}_{o}t+\frac{1}{2}{\left[A\right]}_{o}\left(k_{1}{}^{2}{\left[E\right]}_{o}{}^{2}+k_{1}k_{2}{\left[E\right]}_{o}{\left[A\right]}_{o}\right)t^{2}.$$

Equation (5) is far easier to handle than Equation (3) and is suitable for determining the initial rate (*t* = 0) for the H_2_O_2_ decomposition by catalase in the reaction mixture. In fact, the value for the initial rate (Equation (6)) can be derived directly from the coefficient of the term of degree one of Equation (5).

(6) $$-{\left(\frac{d[A]}{dt}\right)}_{t=0}={[A{]}^{\prime }}_{t=0}\approx -k_{1}{\left[E\right]}_{o}{\left[A\right]}_{o}.$$

**Figure 1:**
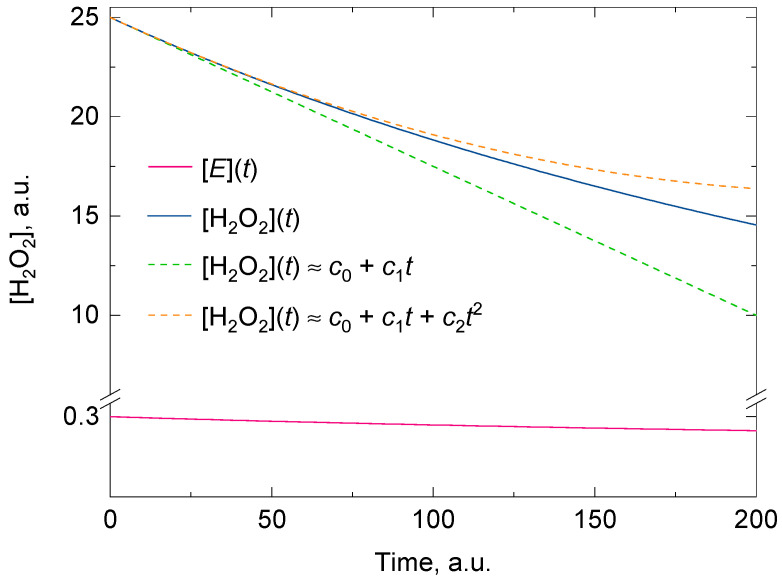
A representative analytical solution of the kinetic model for the two concurrent second-order reactions leading to H_2_O_2_ decomposition by catalase (solid blue line) and its inactivation by H_2_O_2_ (solid red line). The dashed green and dashed orange lines are the polynomials of degree one and two obtained at *t* = 0 from the Taylor series of the exponential analytical solution of [H_2_O_2_]. The polynomial of degree two shows a better overlap with the analytical solution for *t* > 0. The initial conditions for [H_2_O_2_] and [*E*] were 25 and 0.3 arbitrary units and the values for the overall forward rate constants (i.e., *k*_1_ and *k*_2_) of the second-order reactions were 0.01 and 0.00005 arbitrary units, respectively. As *t* approaches infinity, [H_2_O_2_] approaches the value of [H_2_O_2_]*_o_*(1 − *k*_1_[*E*]*_o_*/*k*_2_[H_2_O_2_]*_o_*). Here the condition *k*_2_[H_2_O_2_]*_o_* > *k*_1_[*E*]*_o_* holds. More details are given in the main body of text.

Regardless of whether there is suicide substrate inhibition or not, the fitting of the experimental data for [*A*] with a polynomial of degree two consistently gives a better approximate value for the initial rate than a polynomial of degree one for the same time domain. If a polynomial of degree one was used instead, it would tacitly imply that the kinetic model followed a zero-order reaction and that there was no suicide substrate inactivation in the reaction mixture (two approximations that are questioned when measuring the catalase activity in vitro). Figure 1 shows the goodness of the fit between two polynomials of degrees one and two, and a representative analytical solution of Feuers’ kinetic model.

### 3.2. Linear Dependence of the H_2_O_2_ Decomposition Rate on the Catalase Concentration

The first goal of this study was to determine the initial rate for the H_2_O_2_ decomposition by catalase in vitro without being involved in a complex non-linear fitting procedure. This is, particularly, significant when the researchers are more interested in knowing the values for the initial rates of the biological samples under study rather than the catalase inactivation process, which is inherently linked to the spectrophotometric method. The protocol, here described, has been intentionally developed to measure the initial rate of the H_2_O_2_ decomposition in the first 2 min. This time domain was optimized by trial and error testing to ensure that, firstly, the number of points recorded in the microplate reader for each sample was enough to adequately perform the fitting of the experimental data and, secondly, the accumulation of O_2_ trapped in the detergent bubbles at the top surface of the reaction mixture in the wells of the microplates did not scatter the zenithal measuring beam. The chosen time is insufficient if the researcher’s interest is in accurately determining the inactivation rate constant. In this latter case, the method requires that the H_2_O_2_ decomposition is measured until either catalase is fully inactive or H_2_O_2_ becomes exhausted, which can take tens of minutes. This kind of approach has been used with success to determine the inactivation rate constant of catalase under different experimental conditions including pre-incubation of catalase with H_2_O_2_, temperature, pH or the presence of peroxidatic compounds such as ethanol in the reaction mixture [15,25,26,27,28].

**Figure 2:**
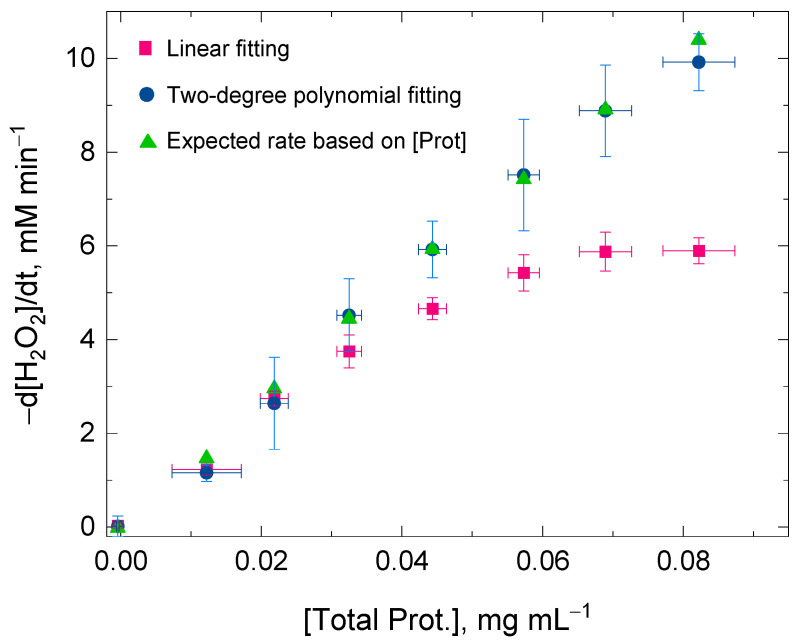
Ratio between the total protein concentration extracted from wheat flag leaves and the initial rate of the H_2_O_2_ decomposition by catalase present in the protein extraction. The values for the initial rates were obtained after linear fitting (red squares) and 2-degree polynomial fitting (blue circles) of the experimental data points recorded in the time domain 0 ≤ *t* ≤ 2 min. The green triangles show the expected values for the initial rate based on the measured protein concentration. The discrepancy between the two fitting procedures was more prominent as the total protein concentration increased in the reaction mixture. A better linear ratio between the initial rate (below 10 mM min^−1^) and the protein concentration was determined when using a 2-degree polynomial fitting. A number of nine replicates using the proposed protocol (Experiment I) were average for each of the eight sample fractions. The total protein concentration for the sample fractions in the reaction mixture was kept below 0.1 mg mL^−1^ and the initial [H_2_O_2_] was always 25 mM. The H_2_O_2_ decomposition rate was followed at 240 nm in a multimodal plate reader.

Figure 2 shows the values for the initial rate of the H_2_O_2_ decomposition by catalase using a linear fitting and a two-degree polynomial fitting for the same time domain (i.e., 2 min). At low protein concentrations, the values for the H_2_O_2_ decomposition rates obtained by either of the two fitting procedures were very similar within experimental error. However, the discrepancy of the values for the initial rates was more evident between the two fitting procedures at high protein concentrations—when the catalase concentration was also high—and the concurrent H_2_O_2_ decomposition and enzyme inactivation reactions were consequently more rapid (Equations (1) and (2)). At high protein concentrations, the changes in absorbance at 240 nm followed a clear curvilinear trend; therefore, a linear fitting was concluded to be inadequate for the kinetic analysis. The values for the H_2_O_2_ decomposition rate using the two-degree polynomial fitting showed a better linearity with protein concentrations, at least until the initial rate of the H_2_O_2_ decomposition reached values close to 10 mM min^−1^ in the reaction mixture (Figure 1). The reason for this discrepancy at high initial rates is methodological and is given in the next section. In conclusion, the two-degree polynomial fitting procedure surpassed the linear fitting procedure and also showed its validity for calculating the initial rate (at *t* = 0) regardless of the progress on the suicide substrate inactivation that catalase might undergo at *t* > 0 during the spectrophotometric measurement.

Moreover, we observed that the experimental conditions chosen to measure the catalase activity according to the method described by Pérez-López et al. [20], satisfies the condition *k*_2_[H_2_O_2_] > *k*_1_[Catalase] because H_2_O_2_ was not completely decomposed and the H_2_O_2_ concentration remained unchanged after several minutes.

### 3.3. Experimental Considerations for a Rapid and High-Throughput Method to Measure the Catalase Activity in Microplate Readers

The second goal of this study was to develop a high throughput protocol to measure the catalase activity, particularly in situations where the researcher has to deal with a large number of biological and technical replicates of various organs or tissues of the living organisms under analysis. These replicates might hold different catalase activities because of the (stress) treatments, the presence of peroxidatic compounds in the reaction mixture or the use of genetically engineered catalases. Because of this variability of experimental conditions, the range of the initial rates is predictably wide; thus, the data points can follow both linear-like and curvilinear trends depending on the catalase activity in each of the microplate wells.

In an attempt to improve the velocity of the protocol, we introduced changes in the way the assay buffer containing H_2_O_2_ was loaded into the wells before the kinetic reading started (Experiment I). In the proposed protocol, the assay buffer was strategically split in two halves to rapidly deposit the half containing H_2_O_2_ (in steps of 100 µL) into the wells without needing to refill the 1.2-mL, eight-channel pipette. This approach notably reduced the loading time of the proposed protocol. For example, a time of 10 s was enough to fill in all the 96 wells of a microplate, while a time of approximately 35 s was required for the standard protocol after refilling the multichannel pipette several times (Figure S1). A two-way ANOVA analysis with interaction between the factors Protocol and Lane (independent variables) was performed to establish whether the difference in the loading time had any statistically significant effect on the measured initial rate of the H_2_O_2_ decomposition by catalase (dependent variable). The assumptions to perform the two-way ANOVA analysis are summarized in the supplementary material (Table S1). Despte the lack of the homogeneity of variance across lanes, the analysis was performed because the lanes had equal sample size and also the number of replicates per lane and protocol (*n* = 30) was higher than 10, two conditions that provide robustness to the ANOVA analysis when homoscedasticity is not fulfilled [29].

The two protocols were compared using all the possible combinations that we could think of using eight different biological samples (Lanes A–H) measured in triplicates up to a maximum of 12 replicates. The two-way ANOVA analysis showed there was a statistically significant interaction between the effects of the factors Protocol and Lane [F(7, 464) = 5.1, *p*-value = 1.4 × 10^−5^], although the effect size (*η*^2^ = 0.003) was small (Table S1). The initial rates were consistently higher (beyond Lane C) in the proposed protocol (Figure S3) and the difference between them was significant after a post-hoc pairwise *t*-test (Table 1). In brief, the split of the assay buffer into two halves is advantageous when the catalase activity is measured simultaneously in several samples and there are no other advanced technical alternatives to speed up the buffer loading with pipetting robots [16,30].

**Figure 3:**
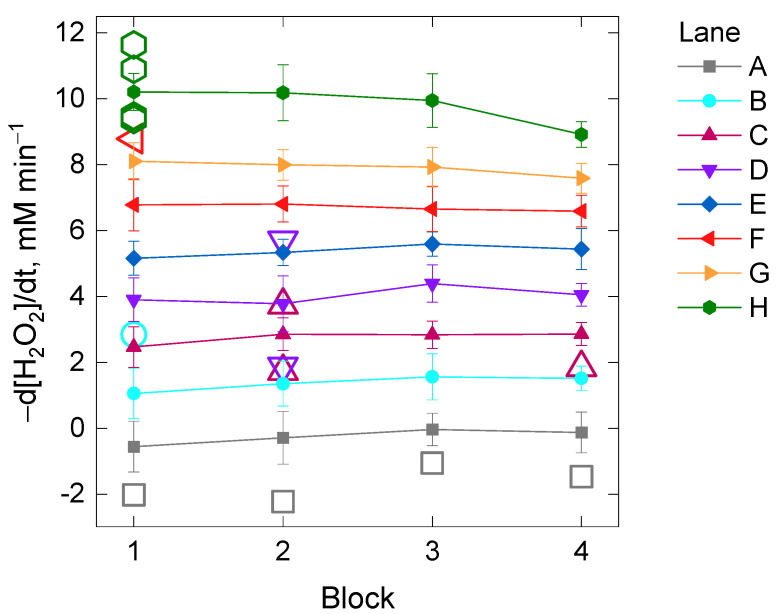
Mean values of the initial rate of the H_2_O_2_ decomposition by catalase in four different blocks of samples differing in the kinetic reading time imposed by their position in the 96-well microplate (Experiment II). Each of the mean values per block from Lane A to H (with increasing total protein concentration) belongs to 15 technical replicates of five repetitions as depicted schematically in Figure S2. The open colored symbols are outliers belonging to the lanes with the same color. See the Material and Methods section for further details.

Additionally, we inspected whether or not the kinetic reading time per cycle could also have a significant impact on the catalase assay (Experiment II). The kinetic reading time exclusively depends on the intrinsic limits of the microplate reader and it varies according to the number of wells (or columns) that are planned to be read in the trial (Figures S1 and S2, *x*-axes). In order to perform this second experiment, the initial rate of the H_2_O_2_ decomposition was compared between four different blocks among which there was an increasing delay time of the starting kinetic reading based on their position in the 96-well microplate (Figure S2). The effect of the two factors Lane and Block and their interaction on the initial rate of the H_2_O_2_ decomposition by catalase was investigated. In spite of finding evidence for heteroscedasticity across the levels of the factor Lane (Table S2), the two-way analysis was performed based on the previous arguments (equal sample size and high number of replicates per Lane and Block, *n* = 15). The two-way ANOVA analysis showed evidence for a statistically significant interaction between the effects of the factors Lane and Block [F(21, 448) = 3.4, *p*-value = 1 × 10^−6^] on the initial rate of the H_2_O_2_ decomposition, although the effect size was small (*η*^2^ = 0.005). A post-hoc pairwise *t*-test between blocks showed that statistically significant differences were found between the block number 4 and the rest when the initial rate of the H_2_O_2_ decomposition by catalase was about 10 mM min^−1^ (i.e., Lane H, adjusted *p*-value < 0.05). Evidence for significant differences was also found between some blocks for 8 mM H_2_O_2_ min^−1^ (i.e., Lane G, *p*-value < 0.05) if corrections for multiple testing were not included (Table S2, Figure 3).

These results allow us to conclude that the number of biological samples that can be analyzed simultaneously with confidence is 32 (assuming three technical per sample) if the initial rate of the H_2_O_2_ decomposition rate does not exceed values beyond 8 mM min^−1^ in any of the biological samples under investigation. Our approach to developing a rapid and high-throughput protocol to measure the catalase activity in vitro is in line with other protocols designed to process, at low cost, a large number of biological samples, in which, for example, the non-enzymatic antioxidant capacity of the leaves of plants or the activity of plant carbohydrate metabolism enzymes are evaluated for environmental adaptation or physiological phenotyping [31,32,33]. Moreover, the spectrophotometric method described by Aebi [5] is used in biological samples from human beings and animals. The catalase activity is employed, for example, as a molecular target for male infertility in human seminal plasma [34] and is measured in erythrocytes and tissues of animals to evaluate the effect of selenium on the prevention of health disorders [35] or the benefits of feed antioxidants in animal health and the quality of animal food products [36]. All this suggests that the rapid and high-throughput protocol, here presented, can also be utilized in the biological matrices of organs or tissues of different living organisms.

## 4. Conclusions

The present study lays down guidelines for the measure of the catalase activity in vitro in a rapid and high throughput protocol. We have chosen catalase because it is an enzyme with unusual kinetic properties showing suicide substrate inhibition and non-substrate saturation, two features that make its kinetic analysis rather complex. After several theoretical considerations, an easy two-degree polynomial fitting is proposed to determine the initial rate of the H_2_O_2_ decomposition by catalase. The proposed protocol includes statistical analyses to make decisions about how many replicates can be measured per trial or which rate limits should be included in the kinetic analysis to avoid any bias in the measure of the catalase activity in vitro. This protocol can be extended to other antioxidant enzyme activity assays following similar guidelines, particularly when the use of a liquid handling robot is not affordable.

## Figures and Tables

**Table 1:** Pairwise *t*-test between lanes of a 96-well microplate containing flag leaf extracts with different protein concentration (see text for further details) ^#^.

Lane	n1	n2	Statistic	Df	*p*-Value	Significance
A	30	30	1.26	29	0.2180	ns
B	30	30	−0.12	29	0.9070	ns
C	30	30	−1.02	29	0.3160	ns
D	30	30	−5.18	29	0.0000	***
E	30	30	−3.70	29	0.0009	***
F	30	30	−3.16	29	0.0040	**
G	30	30	−4.10	29	0.0003	***
H	30	30	−4.76	29	0.0001	***

^#^ The argument for the common standard deviation was set equal to false due to the lack of homogeneity of the variance. Df stands for the degrees of freedom. Significance codes: ‘***’ 0.001 ‘**’ 0.01.

## Data Availability

Data is contained within the article or Supplementary Material.

## Supplementary Materials

The following are available online at https://www.mdpi.com/article/10.3390/antiox11010021/s1, Figure S1. Microplate layout designed to compare the catalase activity in vitro between two protocols differing in the loading time of the assay buffer containing H_2_O_2_; Figure S2. Microplate layout designed to investigate the dependence of the measured initial rate of the H_2_O_2_ decomposition on the well position; Figure S3. Mean values of the initial rate of the H_2_O_2_ decomposition by catalase using two different protocols; Table S1. Analysis of the assumptions to perform a two-way ANOVA analysis in Experiment I; Table S2. Analysis of the assumptions to perform a two-way ANOVA analysis in Experiment II; Table S3. Experiment I data; Table S4. Experiment II data.
