# Supplementary material for: Theoretical and Experimental Considerations for a Rapid and High Throughput Measurement of Catalase In Vitro
Source: Antioxidants (Basel). 2021 Dec 22;11(1):21. doi: 10.3390/antiox11010021 (PMC8773236; doi:10.3390/antiox11010021)
Supplement: Supplementary file 1 [file antioxidants-11-00021-s001.zip › Figure S1_S3 by Arellano.pdf]

Theoretical and experimental considerations for a rapid and high throughput measurement of catalase *in vitro*

Ouardia Bendou [†], Ismael Gutiérrez-Fernández [†], Emilio L. Marcos-Barbero, Nara Bueno-Ramos, Ana I. González-Hernández, Rosa Morcuende and Juan B. Arellano [*]

Department of Abiotic Stress, Institute of Natural Resources and Agrobiology of Salamanca (IRNASA-CSIC), Cordel de Merinas, 40–52, 37008 Salamanca, Spain

[†] These authors contributed equally to this work

[*] Corresponding autor: Juan B. Arellano. E-mail: juan.arellano@irnasa.csic. Phone: +34 923 219 606

Keywords: catalase; hydrogen peroxide; microplate reader; polynomial fitting; suicide substrate

FIGURE S1

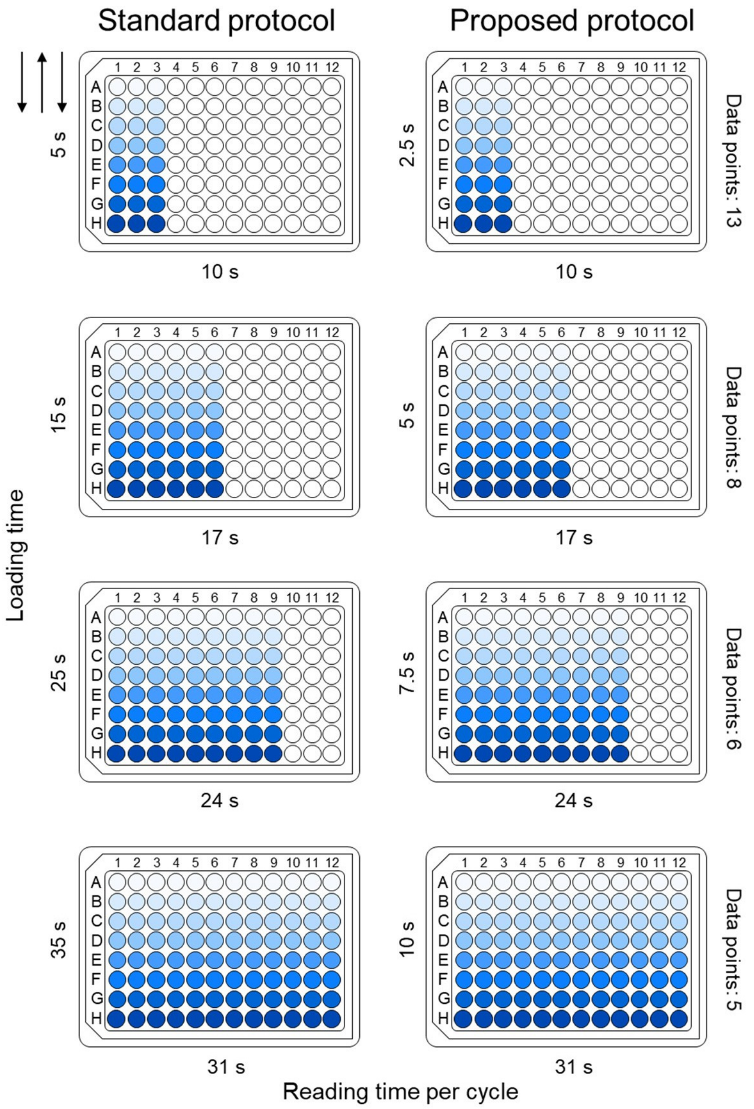

Figure S1. Microplate layout designed to compare the catalase activity *in vitro* between two protocols differing in the loading time of the assay buffer containing H_2O_2. A number of 3, 6, 9 and 12 replicates of 8 samples (forming a linear gradient from low to high total protein concentration) were used to provide robustness to the statistical analysis. The loading time for the H_2O_2 containing assay buffer (the left *y*-axes) and the reading time per cycle (*x*-axes) are shown for both the standard and proposed protocols. The number of data points per cycle recorded in the wells is given in the right *y*-axes. The total number of samples were 480. The straight arrows on the top left hand side corner shows the vertical zigzag pattern for the kinetics reading starting in A1.

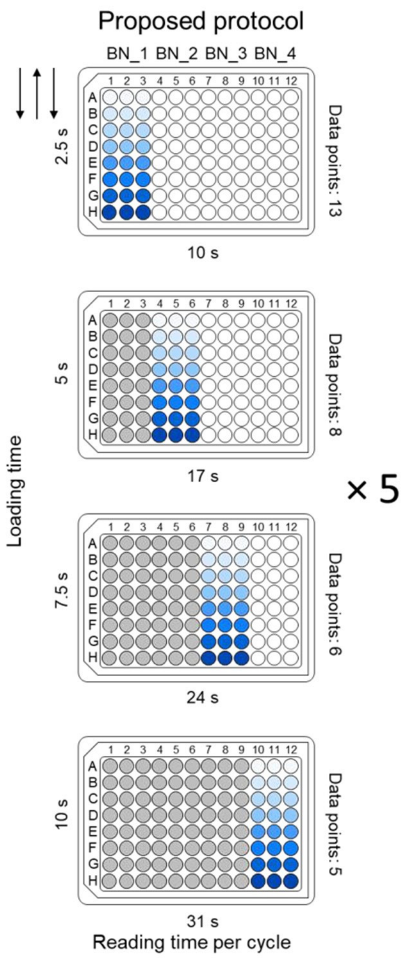

Figure S2. Microplate layout designed to investigate the dependence of the measured initial rate of the H_2O_2 decomposition on the well position. Four blocks in blue (BN_1−4), each containing three replicates per lane of the biological sample, occupy different positions in the microplate to fix different reading times in the trials. Wells in light grey were filled in only with both the H_2O_2-free and the H_2O_2-containing assay buffers to ensure that the reading circles always started in A1 in the four trials. The number of data points per cycle recorded in the wells is given in the right *y*-axes. The experiment was repeated five times (×5) and the total number of samples were 480. The straight arrows on the top left hand side corner shows the vertical zigzag pattern for the kinetics reading.

FIGURE S3

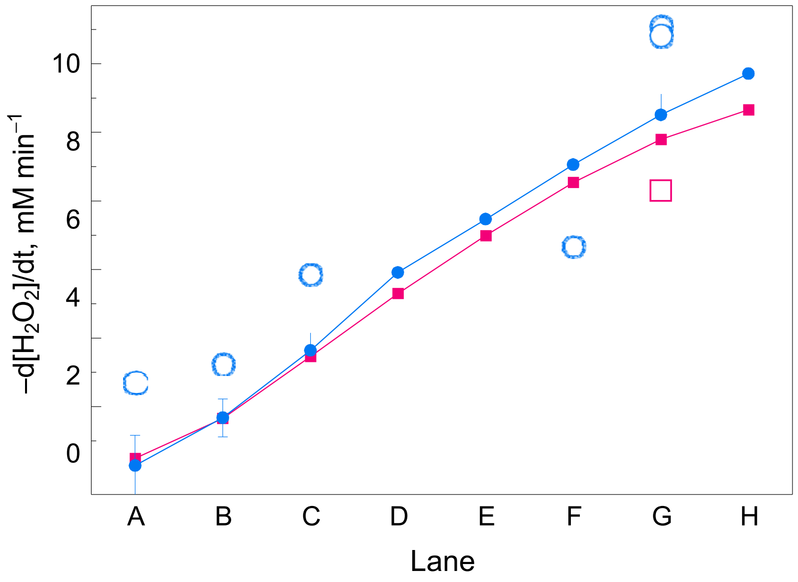

Figure S3. Mean values of the initial rate of the H_2O_2 decomposition by catalase using two different protocols (Experiment I). The main difference between the two protocols resides in the loading time of the assay buffer containing H_2O_2. The solid and open red squares correspond with the mean values of the initial rate and the outliers in the standard protocol and the solid and open blue circles correspond with those in the proposed protocol. The loading time for the trials is lower in the proposed protocol. Each of the mean values from Lane A to H (with increasing total protein concentration) belongs to 30 technical replicates of four combined trials per protocol as depicted schematically in Figure S1. See the Material and methods section for further details.
